# MRI Findings of Two Patients With Hashimoto Encephalopathy

**DOI:** 10.7759/cureus.15697

**Published:** 2021-06-16

**Authors:** Vibeeshan Jegatheeswaran, Michael Chan, Yingming A Chen

**Affiliations:** 1 Department of Medical Imaging, University of Toronto, Toronto, CAN; 2 Department of Diagnostic Imaging, Trillium Health Partners, Mississauga, CAN

**Keywords:** mri, autoimmune, claustrum, hashimoto encephalopathy, sreat, encephalitis

## Abstract

Hashimoto encephalopathy (HE), also known as steroid-responsive encephalopathy associated with autoimmune thyroiditis (SREAT), is a rare type of autoimmune encephalitis that typically presents with cognitive and neuropsychiatric symptoms that resolve with steroids. Positive neuroimaging findings of HE are rarely reported in the literature. We present two cases of HE with abnormal MRI findings, including signal abnormalities in the claustrum, cerebral white matter, and mesial temporal lobes. HE and other forms of autoimmune encephalopathies can often be misdiagnosed as viral encephalopathies. As such detection of subtle neuroimaging findings in the context of suspicious clinical history should prompt further investigations to ensure accurate and timely diagnosis.

## Introduction

Hashimoto encephalopathy (HE), typically presents with a range of symptoms such as cognitive impairment, convulsions, confusion, speech disorder, and delusions [1,2]. HE is associated with the presence of anti-thyroid peroxidase (TPO) and/or anti-thyroglobulin antibodies, and clinical improvement with steroid and immunotherapy is often seen [2-5]. Existing literature on MRI findings of HE reports predominantly normal findings or non-specific white matter changes and infrequently mesial temporal involvement [1,3,4,6,7]. We present two cases of HE with abnormal MRI findings, with some features of this entity not previously described in the literature. High suspicion for HE needs to be maintained when presented with this spectrum of neuroimaging findings to prompt further clinical investigations and to ensure appropriate treatment [1].

## Case presentation

Case 1

**Figure 1:**
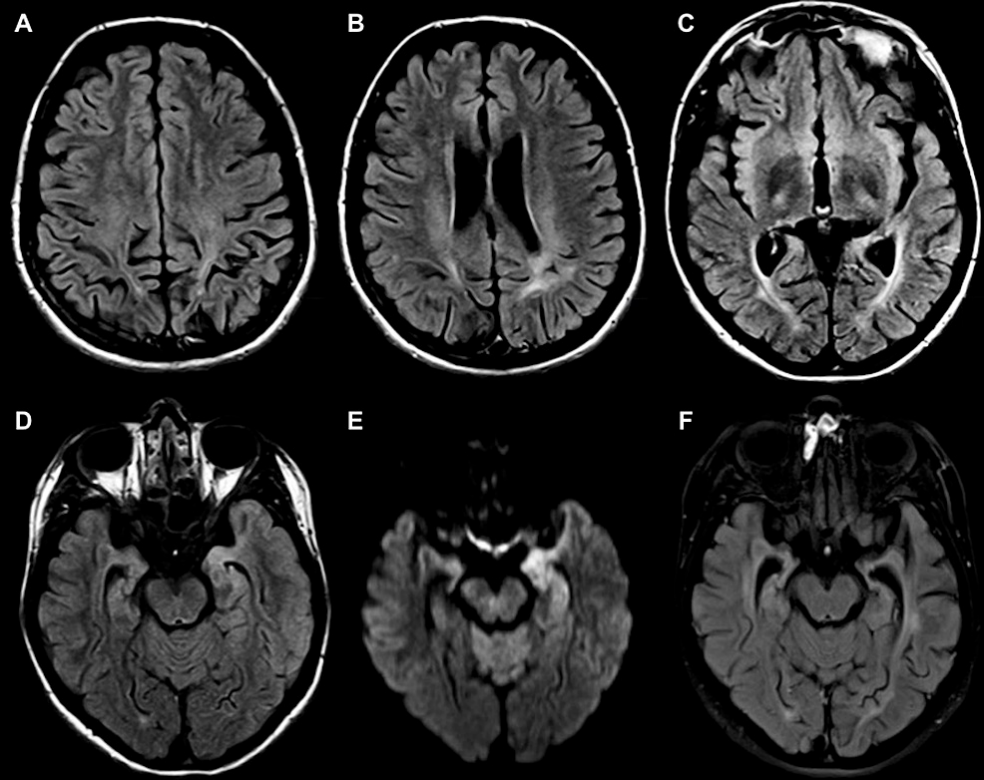
Axial FLAIR images at presentation and two months later Axial FLAIR images at presentation (A-E). There is diffuse brain volume loss out of proportion for age, with ill-defined confluent white matter hyperintensities in bilateral parietal lobes and periatrial regions. The subcortical U-fibers are preserved. There is also asymmetric FLAIR hyperintensity of the left mesial temporal lobe (D) with associated reduced diffusion (E). Axial FLAIR image two months later (F) demonstrate the progression of left mesial temporal atrophy, with a resolution of reduced diffusion (not shown). FLAIR: fluid-attenuated inversion recovery.

A 20-year-old previously healthy female presented with years of nonspecific neurologic symptoms that worsened in the last three to four months including headache, confusion, incoherent speech, and memory difficulties. She was admitted for empiric acyclovir with the suspicion of herpes encephalitis. CT head, CT angiography (CTA), and cerebrospinal fluid (CSF) analysis were all negative. Electroencephalogram (EEG) showed left-sided focal slowing without epileptiform discharges. MRI brain (Figure 1A-1E) revealed diffuse brain volume loss out of proportion for age, and ill-defined bilateral confluent white matter hyperintensities in bilateral parietal lobes and periatrial regions on fluid-attenuated inversion recovery (FLAIR) and T2-weighted images. There was also asymmetric FLAIR hyperintensity of the left mesial temporal lobe with associated reduced diffusion. Ultimately, she was discharged after some clinical improvement without a formal diagnosis.

She presented again two months later with a two-day history of confusion and a tonic-clonic seizure. CSF analysis and CT head were once again negative. New MRI demonstrated resolution of FLAIR hyperintensity in the left mesial temporal lobe, although with progressed atrophy in this region (Figure 1F). Bloodwork was negative for anti-N-methyl-d-aspartate (anti-NMDA) receptor antibodies and other paraneoplastic antibodies (anti-hu, anti-ma, anti-CV2/CRMP5, etc.). Thyroid-stimulating hormone (TSH; 2.54 mIU/L), free T4 (20 pmol/L), T3 (4.5 pmol/L), and anti-TPO antibodies (<10 kIU/L) were all within normal limits. However, antithyroglobulin (266 kIU/L) was elevated and as such a presumptive diagnosis of anti-TPO encephalitis was made. The patient subsequently received 1 g of IV methylprednisolone daily for five days which was later tapered over 16 days to prednisone 60 mg by discharge.

The patient experienced clinical improvement of acute clinical symptoms upon treatment and subsequently discharged. Unfortunately, her symptoms of seizures and confusion recurred four months later and she is currently on levetiracetam and mycophenolate therapy, with clinical stability.

Case 2

**Figure 2:**
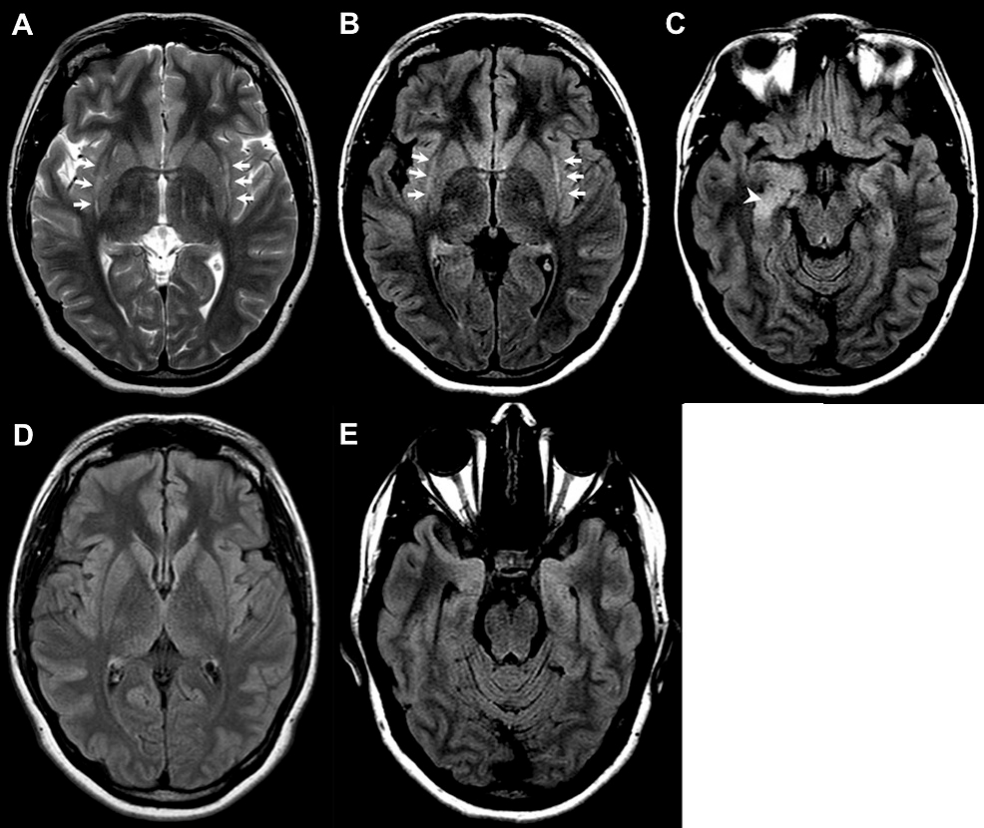
Axial T2-weighted and FLAIR images at presentation and three months later Axial T2-weighted (A) and FLAIR (B, C) images demonstrate bilateral claustral hyperintensities (arrows), and asymmetric hyperintensity of the right mesial temporal lobe (arrowhead). FLAIR images three months later (D, E) showed normalization of claustral and mesial temporal lobe signals. FLAIR: fluid-attenuated inversion recovery.

A 21-year-old previously healthy female presented with a one-day history of confusion, impaired memory, loss of consciousness, and tonic-clonic seizures. CSF analysis was only significant for mild lymphocytic pleocytosis. Viral encephalitis was suspected and she was started on empiric antivirals and levetiracetam for seizures. CSF was ultimately negative for the herpes simplex virus (HSV). An initial CT head was negative, and an EEG showed potentially epileptogenic activity in right central and temporal recordings. MRI (Figure 2A-2E) demonstrated symmetric signal abnormalities in the bilateral claustrum, as well as the right mesial temporal lobe. 

Antinuclear antibody (ANA), c-antineutrophil cytoplasmic antibody (c-ANCA), and p-antineutrophil cytoplasmic antibody (p-ANCA) were negative. TSH was low at 0.31 mIU/L and free T4 and T3 were 14 pmol/L and 2.6 pmol/L, respectively. Anti-TPO and antithyroglobulin (Tg) antibodies were elevated at 163 kIU/L and TgAb 338 kIU/L, respectively, and a presumptive diagnosis of HE was made. The patient was started on 1 g of IV methylprednisolone as well as one dose of IVIG. There was a significant clinical improvement by day 10 of treatment and she was discharged on 60 mg prednisone. Her speech and cognition returned to near baseline three months later, with follow-up MRI demonstrating almost complete interval resolution of the claustral and right hippocampal signal abnormalities.

## Discussion

We present two cases of HE with positive MRI findings before and after treatment with steroid therapy. The first case displayed diffuse volume loss and bilateral confluent white matter hyperintensities, which persisted post-steroid therapy despite clinical improvement. Consequently, the imaging findings likely reflect prolonged brain inflammation secondary to undiagnosed long-standing anti-TPO autoimmune encephalitis. The second case demonstrated reversible bilateral claustral hypersignal, a finding not previously described in HE but has been described in autoimmune encephalitis (AE) [8].

The clinical diagnosis of HE is made by clinical findings of encephalopathy, and abnormal elevation of thyroid antibodies, including antithyroglobulin or anti-TPO, the latter of which is elevated in almost all cases [9]. Titers of plasma antithyroid antibodies do not correlate well with the severity of encephalopathy and may remain elevated after treatment and clinical improvement [5]. Furthermore, over 40% of reported cases of HE are euthyroid at the time of diagnosis (as seen in both our patients), although most have or develop Hashimoto’s thyroiditis [10].

Existing literature demonstrates the variable nature of HE with regards to neuroimaging findings [1,3,4,6,7,11,12]. Castillo et al. found abnormal diffuse white matter signal abnormalities in 4/19 (26%) patients with a clinical diagnosis of HE [1] out of proportion for age. Matsunaga et al. presented 5/12 HE patients with non-restricting diffuse white matter hyperintensities, a few of which fluctuated with clinical disease course [4]. More punctate diffusion restricting white matter lesions have also been described in acute presentations of HE in patients without cerebrovascular risk factors, and suggest underlying inflammatory vasculitic phenomenon causing cytotoxic microvascular ischemia [4,13]. In general, diffuse white matter changes in relatively young patients suggest a cause other than chronic ischemic changes and have a differential diagnosis including autoimmune encephalitis, cerebral vasculitis, neurodegenerative diseases, and slow viral infections such as subacute sclerosing panencephalitis (SSPE).

The presence of reversible claustral signal abnormalities in the second patient has been reported in the context of AE. Steriade et al. presented four patients with autoimmune epilepsy and/or encephalitis with significant claustral FLAIR hyperintensities that normalized despite the persistence of epilepsy [8]. One proposed hypothesis for claustral involvement is that the claustrum serves as a connection point between epileptogenic cortices within the limbic system which are activated in autoimmune encephalitis [8,14]. Both our cases also had mesial temporal signal abnormalities that resolved on a follow-up MRI scan. The mesial temporal lobe is known to be involved in AE [15], and more specifically in HE patients who experienced seizures [11,12]. As many AE and HE patients often present with seizures, it is unclear if the mesial temporal findings are due to chronic inflammation or peri-ictal state.

Other imaging manifestations of HE not seen in our cases include reversible cytotoxic lesion of the corpus callosum splenium, and acute ischemic changes involving grey matter [16,17]. Chen et al. showed a case of multifocal acute infarction involving bilateral caudate nuclei, frontal lobes, and internal capsule; and Song et al. described a patient with basal ganglia ischemia [17,18]. The underlying pathophysiology for these findings remains unclear but presumed due to an inflammatory vasculitic process.

A defining characteristic of HE is the reversibility with immunosuppressive therapy [2,4,5], which has been shown to resolve acute neuroimaging abnormalities [1,4,16] along with clinical symptoms. Early diagnosis and intervention improve clinical and imaging outcomes, and clinical relapse has been shown to correlate with imaging exacerbation [4,17]. First-line therapy is corticosteroids with approximately 50% of cases being completely responsive [9]. A small subset of patients who are steroid-resistant may require additional therapy with other immunosuppressive medications such as mycophenolate, or treatment with plasma exchange and IVIG. Levetiracetam has also been shown to be an effective treatment for HE due to its anti-seizure and anti-inflammatory effects [19] and was used successfully in our first patient who became resistant to steroid therapy.

Castillo et al. also found that HE was misdiagnosed in all 20 of the patients they studied [1]. Viral encephalitis was the most common misdiagnosis they found (5/20). In both cases presented here, viral encephalitis was initially suspected, and both patients were treated with antivirals. Oyanguren et al. found that herpes encephalitis was more likely to show MRI abnormalities than AE (100% to 60%). Furthermore, they found that when the temporal lobe is involved, herpes encephalitis demonstrated diffuse involvement, whereas temporal lobe abnormalities in AE patients often localized to the mesial region [15]. Though it is vital to empirically treat suspected cases of viral encephalitis, HE should be kept on the differential especially if CSF is negative or deemed unlikely for an infectious cause.

## Conclusions

There is a wide range of reported neuroimaging abnormalities in HE. We present two cases of HE that demonstrate this fluctuation and variability. HE is often misdiagnosed and can be mistaken for other autoimmune encephalitides or viral encephalitis. As such it is important to understand the neuroimaging features that can potentially differentiate these entities. MRI is valuable in the setting of this disease not just for diagnosis but to evaluate evolution and resolution of the abnormalities.

## References

[1] Castillo P, Woodruff B, Caselli R (2006). Steroid-responsive encephalopathy associated with autoimmune thyroiditis. Arch Neurol.

[2] Laurent C, Capron J, Quillerou B, Thomas G, Alamowitch S, Fain O, Mekinian A (2016). Steroid-responsive encephalopathy associated with autoimmune thyroiditis (SREAT): characteristics, treatment and outcome in 251 cases from the literature. Autoimmun Rev.

[3] Graus F, Titulaer MJ, Balu R (2016). A clinical approach to diagnosis of autoimmune encephalitis. Lancet Neurol.

[4] Matsunaga A, Ikawa M, Kawamura Y (2019). Serial brain MRI changes related to autoimmune pathophysiology in Hashimoto encephalopathy with anti-NAE antibodies: A case-series study. J Neurol Sci.

[5] Olmez I, Moses H, Sriram S, Kirshner H, Lagrange AH, Pawate S (2013). Diagnostic and therapeutic aspects of Hashimoto's encephalopathy. J Neurol Sci.

[6] Kothbauer-Margreiter I, Sturzenegger M, Komor J, Baumgartner R, Hess CW (1996). Encephalopathy associated with Hashimoto thyroiditis: diagnosis and treatment. J Neurol.

[7] Kelley BP, Patel SC, Marin HL, Corrigan JJ, Mitsias PD, Griffith B (2017). Autoimmune encephalitis: pathophysiology and imaging review of an overlooked diagnosis. AJNR Am J Neuroradiol.

[8] Steriade C, Tang-Wai DF, Krings T, Wennberg R (2017). Claustrum hyperintensities: a potential clue to autoimmune epilepsy. Epilepsia Open.

[9] Zhou JY, Xu B, Lopes J, Blamoun J, Li L (2017). Hashimoto encephalopathy: literature review. Acta Neurol Scand.

[10] Schiess N, Pardo CA (2008). Hashimoto's encephalopathy. Ann N Y Acad Sci.

[11] McCabe DJ, Burke T, Connolly S, Hutchinson M (2000). Amnesic syndrome with bilateral mesial temporal lobe involvement in Hashimoto's encephalopathy. Neurology.

[12] Ishitobi M, Yoneda M, Ikawa M, Matsunaga A, Ueno K, Kimura H, Wada Y (2013). Hashimoto's encephalopathy with hippocampus involvement detected on continuous arterial spin labeling. Psychiatry Clin Neurosci.

[13] Grommes C, Griffin C, Downes KA, Lerner AJ (2008). Steroid-responsive encephalopathy associated with autoimmune thyroiditis presenting with diffusion MR imaging changes. AJNR Am J Neuroradiol.

[14] Crick FC, Koch C (2005). What is the function of the claustrum?. Philos Trans R Soc Lond B Biol Sci.

[15] Oyanguren B, Sánchez V, González FJ (2013). Limbic encephalitis: a clinical-radiological comparison between herpetic and autoimmune etiologies. Eur J Neurol.

[16] Bohnen NI, Parnell KJ, Harper CM (1997). Reversible MRI findings in a patient with Hashimoto's encephalopathy. Neurology.

[17] Chen N, Qin W, Wei C, Wang X, Li K (2011). Time course of Hashimoto's encephalopathy revealed by MRI: report of two cases. J Neurol Sci.

[18] Song YM, Seo DW, Chang GY (2004). MR findings in Hashimoto encephalopathy. AJNR Am J Neuroradiol.

[19] Wong LC, Freeburg JD, Montouris GD, Hohler AD (2015). Two patients with Hashimoto's encephalopathy and uncontrolled diabetes successfully treated with levetiracetam. J Neurol Sci.

